# Exploring the Underlying Causes of Chinese Eastern Star, Korean Sewol, and Thai Phoenix Ferry Accidents by Employing the HFACS-MA

**DOI:** 10.3390/ijerph17114114

**Published:** 2020-06-09

**Authors:** Xiaolong Wang, Boling Zhang, Xu Zhao, Lulu Wang, Ruipeng Tong

**Affiliations:** School of Emergency Management and Safety Engineering, China University of Mining and Technology-Beijing, Beijing 100083, China; WXLaqkx@163.com (X.W.); zbl9856@163.com (B.Z.); zhaoxu4016@163.com (X.Z.); wanglulu1027@126.com (L.W.)

**Keywords:** maritime safety, accident prevention, human factors, HFACS-MA

## Abstract

Maritime safety is a significant topic in the maritime industry since the numerous dangers at sea could lead to loss of property, environmental pollution, and even casualties. Existing research illustrates that human factors are the primary reasons of maritime accidents. Indeed, numerous maritime accidents can be classified into different types of human factors. In this context, the Human Factors Analysis and Classification System for Maritime Accidents (HFACS-MA) model is introduced in this paper. The HFACS-MA framework consists of five levels, complying with the core concepts of HFACS and the guiding principles of the International Maritime Organization (IMO). Based on the five levels of the framework, this research explores the underlying causes of Chinese Eastern Star, Korean Sewol, and Thai Phoenix accidents, and a comparative analysis is conducted. The analysis demonstrates the utility of applying the HFACS-MA model to the maritime industry, and the results emphasize the importance of the following categories: legislation gaps, organizational process, inadequate supervision, communication (ships and VTS), decision errors, and so on. Consequently, the research enables increased support for HFACS-MA and its application and provides valuable information for safety management and policy development in the maritime industry at different levels.

## 1. Introduction

The maritime industry is the global economic lifeblood, transporting about 90% of global trade, so maritime safety is a vital factor for the sustainable development of international economics and trade [1]. The maritime industry includes several components such as crew members, maritime regulatory bodies, ships, ship owners, and classification societies, and these components have different effects on safety performance [2]. Maritime safety has always been an important aspect for the maritime industry because there are numerous dangers that can drastically lead to casualties, environmental pollution, as well as tremendous property damage [2,3,4]. According to the literature review of accident analysis in the maritime industry, most maritime accidents are caused by human errors [5,6,7]. Therefore, it is essential to understand human error and organizational factors contributing to accidents for effective management and policy development in the maritime industry [8].

The International Maritime Organization (IMO), whose intention it is to create a fair and effective regulatory framework for the maritime industry in order to decrease human errors, has issued a number of maritime conventions. The four main maritime regulatory conventions are International Convention for the Prevention of Marine Pollution from Ships (MARPOL), Convention on the International Regulations for Preventing Collisions at Sea (COLREG), International Convention on Standards of Training, Certification and Watchkeeping for Seafarers (STCW), and International Convention for Safety of Life at Sea (SOLAS) [2]. In particular, the SOLAS convention refers to numerous requirements for maritime accident prevention aiming to reduce human errors and improve safety awareness [9,10,11,12]. Furthermore, the SOLAS convention provides the minimum safety standards for the construction, machine, equipment, operation, and maintenance of ships, and hence SOLAS is regarded as the most significant convention involving the safety of ships [11]. According to the ship accident data investigated by Tzannatos and Kokotos, the number of accidents is significantly reduced after the International Safety Management (ISM) Code was implemented in 1998 [13,14]. Although the maritime authorities have adopted a set of regulations and rules to regulate safety standards, these regulations or rules are not fully effective, and consequently, maritime accidents caused by human errors continue to happen and have not yet fallen to an acceptable level [15,16]. In order to reduce maritime accidents as far as possible, it is necessary to pay attention to the types of human errors [17].

This paper takes three catastrophic maritime accidents as examples to identify different kinds of human and organizational factors in the maritime industry: the Chinese Eastern Star, the Korean Sewol, and the Thai Phoenix accidents. There are several reasons for selecting the three accidents. Firstly, all the three maritime accidents happened in recent years and resulted in loss of property and heavy casualties. Secondly, the three accidents occurred in China, Korea, and Thailand, respectively, and they are all located in the Asian region with similar social contexts. After the capsizing of Eastern Star and Sewol, several scholars conducted detailed analysis on the accident causes [18,19,20,21]. However, few researchers conducted a comparative analysis on the causes of the three major maritime accidents. Therefore, this paper discusses an in-depth analysis of the underlying causes of the three maritime accidents that happened in three different countries, attempting to explore the similarities and differences about the accident causes under the similar social context. The investigation data of this paper are based upon accident investigation reports, some news articles, and communication with relevant scholars. This study is expected to make a contribution to safety management in the maritime industry. In addition, the abbreviations involved in this paper are presented in Table 1.

## 2. Brief Introduction of Eastern Star, Sewol, and Phoenix Ferry Accidents

### 2.1. The Eastern Star Accident in China

On 1 June 2015 (Beijing time), the Eastern Star, which is owned by the CESC, departed from Nanjing City and was sailing to Chongqing City. At 21:30, the Eastern Star encountered a squall line system accompanied by strong convective weather, tornadoes, as well as torrential rain. Influenced by strong storms, the Eastern Star capsized in a short time when the ship navigated in the Jianli waters affiliated to the Yangtze River of China. The Eastern Star is a large-scale travel ship that was initially built by the Chuandong shipyard in 1994 and renovated in 1997, 2008, and 2015, respectively. The passengers on board were mostly old people from a travel agency named Sunset Red and most of them were sleeping when the accident happened. The accident caused 442 deaths and was deemed to be the most serious maritime accident since 1949 [21]. The severe weather is recognized to be the leading cause of the accident according to the accident investigation report.

### 2.2. The Sewol Accident in South Korea

At 08:58 on 16 April 2014, the Sewol with 476 persons capsized on the way from Incheon port to Jeju Island, and 325 out of the 476 passengers were high school students who were going on a school trip. As an 18-year-old Japanese ship, the Sewol was purchased by the Cheonghaejin Marine Company in 2012. Subsequently, the Cheonghaejin made a series of modifications in a Korean yard to boost capacity in 2013, resulting in the Sewol’s instability [22]. When leaving the port, the Sewol loaded 2142.7 tons of cargo, which was twice the loading limit (987 tons) [20]. During the navigation, Sewol lost its balance when the inexperienced helmsman made a sharp turn. When the captain communicated with VTS for help, the captain made wrong decisions, specifically, the passengers were instructed to stay in the cabins. When the captain told passengers to evacuate, it was too late—at this time the captain and crew members abandoned the ship and fled. The accident caused 304 deaths, and most of the casualties were high school students. The disaster is recognized to be a national disaster in South Korea [18,23].

### 2.3. The Phoenix Accident in Thailand

On 5 July 2018, the Phoenix went to sea illegally in spite of the weather warning. At 17:45, the ship encountered a severe storm on the way back to Phuket and capsized near Coral Island, resulting in 47 deaths [24]. The severe weather was recognized to be the direct cause of the accident [25]. In addition, the crew members did not promptly remind tourists to pay attention to safety and take necessary measures, and many tourists still stayed in the cabin when the ship was about to tilt instead of running to the board, and even many passengers did not wear the lifejacket when the ship was sinking. In addition, the VTS at chalong dock did not receive the distress signal, and the location of the Phoenix disappeared from the positioning system, which may be caused by the severe storm. Moreover, the Marine Office did not take the initiative to contact the Phoenix, which delayed the accident rescue. The Phoenix ferry accident was considered to be the worst travel accident in Thailand’s history.

### 2.4. Overview of the Three Accidents

The overview of the three maritime accidents is presented in Table 2.

## 3. Methodology

It is significant to choose an accident model because the choice of the model determines the methods of data collection, and the conclusions and the preventive measures could be different. In other words, ‘what-you-look-for’ determines ‘what-you-find’, and ‘what-you-look-for’ depends on the selected method or model employed in accident analysis [11,26]. Therefore, it is significant to select a model before conducting an accident investigation. Due to the characteristics of reliability and effectiveness, Human Factors Analysis and Classification System (HFACS) was considered to be one of the preferred models when identifying the human errors and organizational factors as soon as gathering accident information [27].

**Figure 1:**
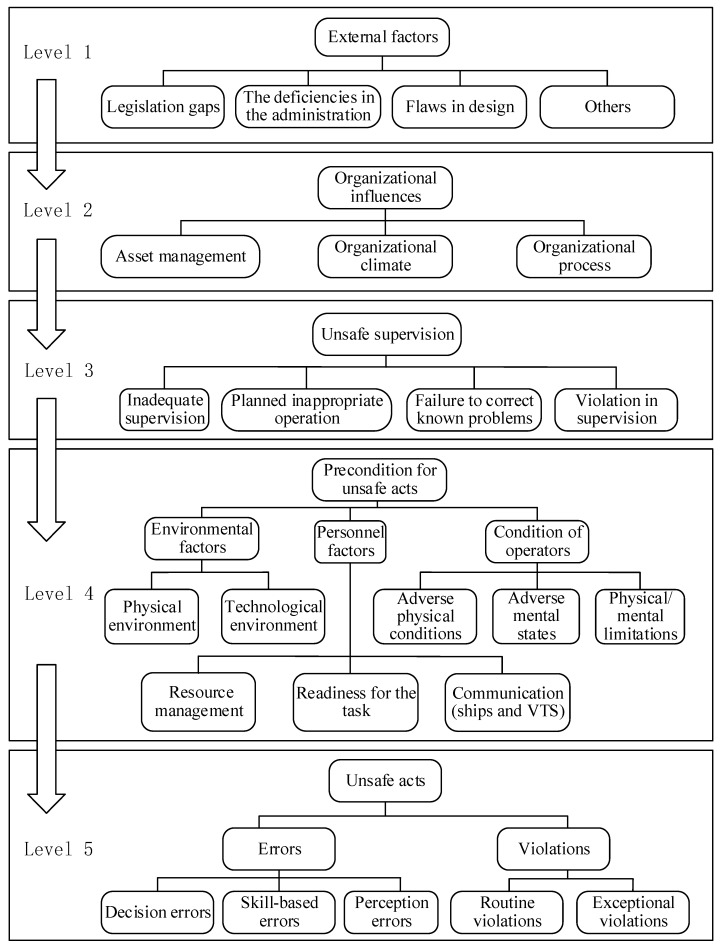
The overview of the Human Factors Analysis and Classification System (HFACS-MA) framework.

HFACS, which is recognized as an epidemiological model, enables one to analyze the visible and underlying causes [28]. In 1997, the HFACS was initially developed by Shappell and Wiegmann on the basis of the Swiss Cheese Model to investigate and analyze the data of the military aviation accidents. The HFACS was gradually applied in other domains, such as civil aviation [29,30,31], maritime industry [32,33], coal mining [34,35,36], medicine [37,38,39], construction industry [40], and railway [41]. However, when applied to other domains, the adapted HFACS model has been proposed by analysts for the purpose of adapting to the characteristics of certain domains. In the maritime domain, there exist many national rules, international agreements, and regulations to ensure the operation of maritime industry. Therefore, according to the features of maritime accidents, this research employs the Human Factors Analysis and Classification System for Maritime Accidents (HFACS-MA) framework to analyze the maritime accidents. The framework is composed of five levels, complying with the core concepts of HFACS and the guiding principles of IMO. In addition, the HFACS-MA model has wide applicability for maritime accidents, including the ship grounding, collisions, and grounding accidents; thus, HFACS-MA framework is a universal model for maritime accidents. Furthermore, HFACS-MA is not limited to management and regulatory mechanisms of countries according to the levels of the framework. Therefore, this paper adopts the HFACS-MA framework to analyze the three maritime accidents. Figure 1 presents the overview of the HFACS-MA framework, and Table 3 provides a concise description of categories involved in the five levels of the framework.

With respect to the HFACS-MA framework, some changes have been made by comparing it with the original HFACS model. Firstly, the level of external factors is added into the HFACS-MA framework to capture the contributing factors that go beyond the scope of the organizational level, the additional level follows the application of HFACS model in the mining industry and railroad industry [34,41], and is modified according to the situation of maritime industry. The external factors level is grouped into four subcategories: legislation gaps, the deficiencies in the administration, flaws in design, and others. Legislation gaps seek to identify the defects of the current rules or guidelines that provide guidance for the maritime industry and the related organizations. The Herald disaster that happened in 1987 suggested that legislation gaps were the main contributing factors for the maritime accidents [28]. The deficiencies in the administration emphasize the failures that relevant authorities fail to perform their duties to ensure the implementation of the existing rules or guidelines, and these defects are recognized to be the contributing factors of some accidents. When identifying the factors related to the weak system design, the flaws in design should be paid more attention and added to the HFACS framework [42]. These defects are often observed and are deemed as the causation factors of an accident, for instance, weak considerations for ergonomics. In addition, some factors that are illustrated in the accident investigation report are categorized as the ‘others’ subcategory since they do not belong to the categories above, for instance, an improper installation at the shipyard falls into this category. Secondly, the category of personal factors is composed of communication (ships and VTS), resource management, and readiness for the task. In the maritime industry, communication among ships and with VTS is crucial, and poor communication could result in lack of coordination [27]. Therefore, the communication (ships and VTS) is added to the HFACS-MA framework. The category of resource management is composed of teamwork, communication, and coordination. When the condition of the ship changes, the team will act accordingly and make use of relevant technical, human, and material resources to ensure voyage safety. The category of readiness for the task includes the physical or mental factors that result in failure to be ready for performing the task, for instance, drug use falls into this category.

## 4. Application and Discussion

In this section, the utility of applying the HFACS-MA model to the three maritime accidents is demonstrated. In order to extract the causation factors accurately, four safety experts are invited to analyze the three maritime accidents based on the HFACS-MA framework. Each safety expert needs to extract the causation factors independently according to the accident investigation report and some news articles, and an in-depth discussion about the causation factors is conducted among the four safety experts. When the causation factors are determined, they are classified into the appropriate level based on experts’ experience, and a comparative analysis is carried out. Practical implications and limitations are discussed.

### 4.1. Cause Analysis of the Eastern Star Accident in China

**Figure 2:**
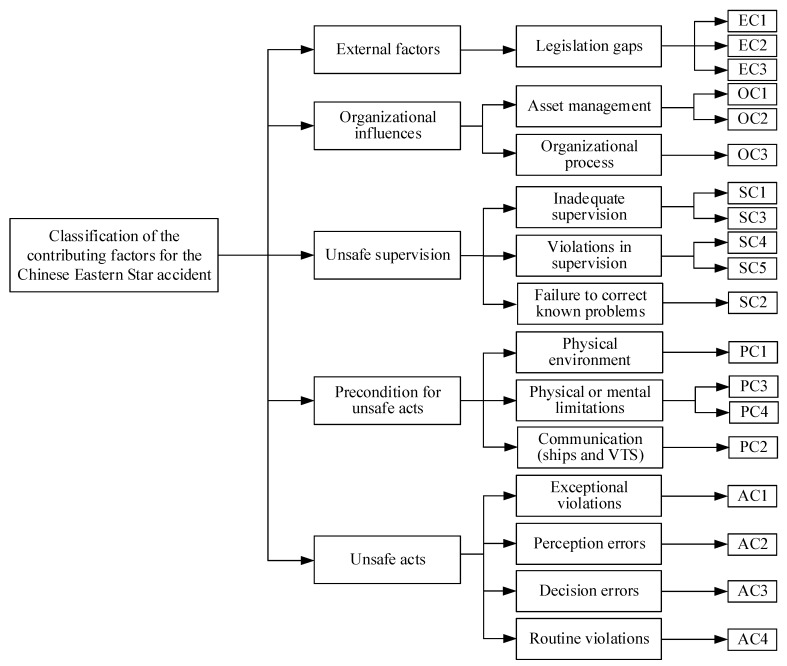
The classification of the contributing factors for the Chinese Eastern Star accident.

In terms of the Chinese Eastern Star accident, 19 accident causation factors are identified according to experts’ experience, and the accident causation factors are listed in Table 4. The classification of the contributing factors for the Chinese Eastern Star accident is shown in Figure 2.

### 4.2. Cause Analysis of the Sewol Accident in South Korea

**Figure 3:**
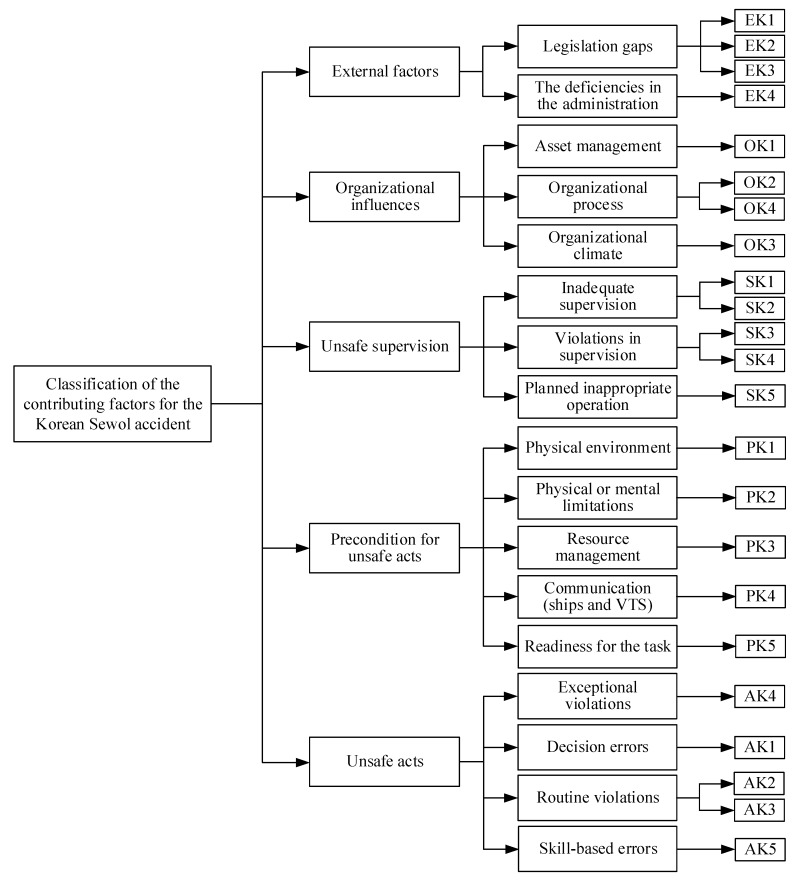
The classification of the contributing factors for the Korean Sewol accident.

With respect to the Sewol accident in South Korea, the disaster is the result of neglecting safety by government, supervising authorities, Chonghaejin, and the crew members [18]. Based on the 5 levels of the HFACS-MA model, 23 causation factors of the Sewol accident are identified, as listed in Table 5, and the classification of the contributing factors for the Korean Sewol accident is presented in Figure 3.

### 4.3. Cause Analysis of the Phoenix Accident in Thailand

**Figure 4:**
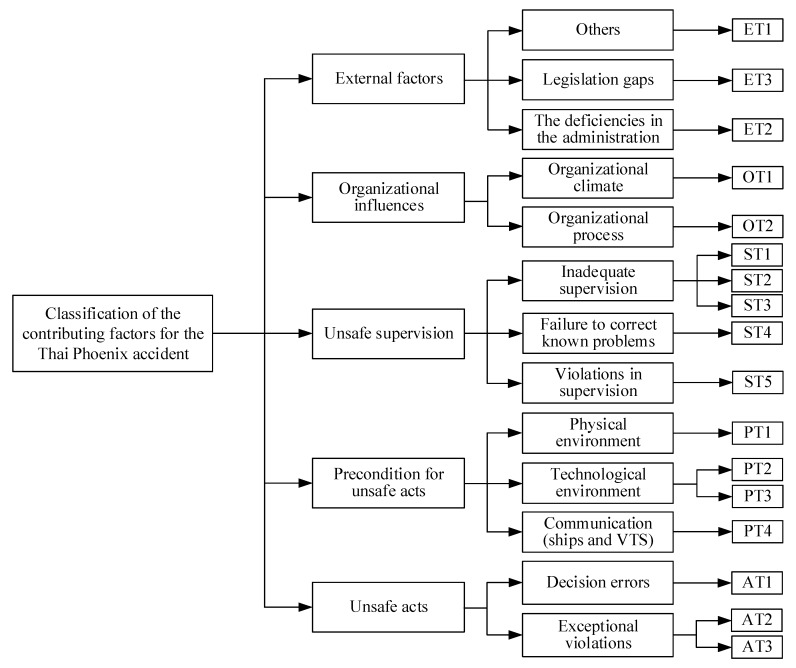
The classification of the contributing factors for the Thai Phoenix accident.

Regarding the Phoenix ferry accident, 17 causation factors are identified based on the five levels of the HFACS-MA framework. Table 6 presents the detailed list of the causation factors, and the classification of the contributing factors is shown in Figure 4.

### 4.4. Comparative Analysis about the Causes of Three Accidents

**Figure 5:**
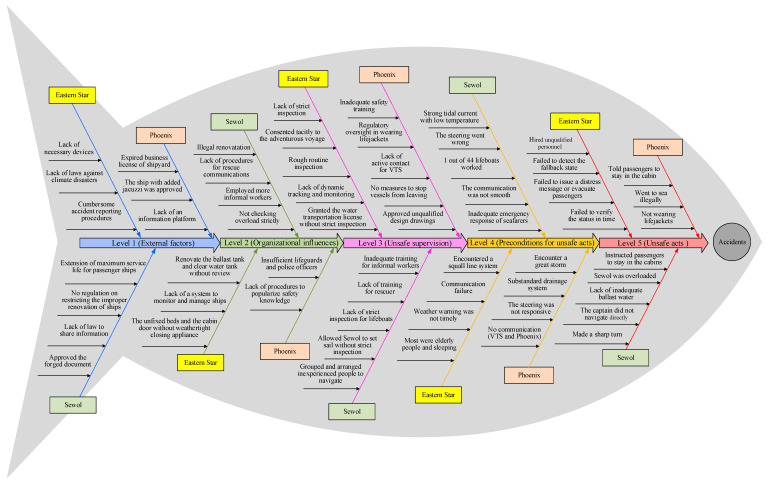
The adapted fishbone diagram about the contributing factors of three ferry accidents.

Based on the cause analysis of the three accidents using the HFACS-MA framework, comparative analysis about the causes of three accidents is conducted, and the analysis results are presented in the form of an adapted fishbone diagram, which is shown in Figure 5. In the adaptation of the fishbone diagram, the “eye” of the fish represents the occurrence of accidents, and the main bone, which is located in the diagram axis, is composed of five arrows with different colors; from the beginning of the diagram axis to the end, the five arrows are in accordance with the five levels of the HFACS model. For the fishbone diagram, we employed the causation factors of the three accidents to fill in the bones of the fish. In addition, the demographics of the causation factors associated with the three accidents are computed and presented in Table 7. In terms of the distribution of the causation factors, there exist similarities and differences in the three accidents. As seen in Table 7, the level of unsafe supervision is the most frequent level for the three accidents, followed by the level of precondition for unsafe acts and unsafe acts. In the following, we make a discussion from the five levels of the HFACS-MA framework.

Regarding the level of external factors, legislation gaps are the primary category associated with the three accidents, especially in the Eastern Star accident and Sewol accident, as seen in Table 7. The Eastern Star accident illustrated that China lacked a specific law to enhance the ability of defending against meteorological disasters; additionally, the necessary devices required by IMO, such as the Global Maritime Distress and Safety System (GMDSS), were not mandatory for Chinese vessels because no corresponding regulations were required by Chinese law. Regarding the Sewol accident, the maximum allowable age of passenger ships was modified to 30 years rather than the previous 20 years by law [18]. The Ships Safety Act in Korea did not limit the improper renovation of ships, and the KSA and KCG did not realize the new capacity limit after renovation. Additionally the Korean laws did not require the supervising authorities to share information effectively. Therefore, it is important to establish and improve the related legislation for maritime safety.

At the level of organizational influences, Table 7 indicates that organizational process is the most commonly organizational factor for the three accidents, which is in line with the existing findings [31]. The CESC did not establish the system to monitor and manage the Eastern Star, the KCG lacked the standard procedures to communicate with Sewol when they received the distress call, and the TC BLUE DREAM company lacked the basic procedures to popularize safety knowledge when the passengers boarded the Phoenix. Therefore, it is important to establish the corresponding procedures for maritime safety, such as safety management system and rescue communications. In addition, the Eastern Star accident and Sewol accident emphasize the defects in the asset management. Both the Eastern Star and Sewol were illegally renovated, which could make the ship easier to capsize. Therefore, the safety limits of the ship should follow the ship’s original design, if the ship is renovated properly, the risk assessment should be conducted routinely to guarantee safety.

As for the level of unsafe supervision, the most frequent categories involved in the three accidents are inadequate supervision and violations in supervision. The efficiency of organizational supervision is one of the influencing factors for the system safety and performance [35]. Figure 5 and Table 7 illustrate that the inadequate supervision is the most frequently supervisory factor associated with the three accidents, which is in accordance with the previous findings [31]. Taking the Eastern Star for example, CSIB and WSIB did not strictly carry out the ship inspection according to the requirements, and they did not find that CESC changed the ballast tank and water tank without authorization; the cabin doors were not equipped with weathertight closing appliance; the bed was not fixed tightly. Furthermore, the three accidents verified the importance of safety training. Taking the Sewol accident for example, more than half of the crew members on the Sewol were informal workers, and the Cheonghaejin company did not provide adequate training for them [18]; thus, the crew on board did not provide immediate and accurate actions during emergency; additionally, the rescue workers provided poor initial rescue operation due to the lack of adequate training and climbing equipment [19]. In addition, violations in supervision were also key contributing factors in the three accidents, which increased the possibility of accidents to some extent. In the safety critical domains, the guidelines of enhancing supervision emphasize the importance of minimizing the violations in supervision.

With respect to the level of precondition for unsafe acts, it was the most vulnerable level since the faults in the ship’s hardware could be inspected at a glance. Table 7 demonstrates that physical environment and communication (ships and VTS) are the primary categories associated with the three accidents. In terms of the environmental factors, the physical environment factors featured remarkably in the three accidents, specifically, Eastern Star, Sewol, and Phoenix encountered the inclement weather such as the squall line system, which made the ships be under unsafe conditions. In addition, communication among ships and with VTS failed to provide effective support for coordination and accident rescue, which increased the accident losses. Previous research has indicated that the communication among ships and with VTS is essential, which can provide timely information for accident rescue to reduce accident losses.

As the Swiss Cheese Model (SCM) suggests, the defects in the external factors, organizational influences, unsafe supervision, and precondition for unsafe acts result in unsafe acts [46]. Therefore, we should focus our efforts on some critical categories at these levels to reduce unsafe behavior. Table 7 demonstrates that the decision errors and exceptional violations were the main categories at the level of unsafe acts. Decision errors in the three accidents mainly refer to the failure in evacuating passengers in time, and violations are usually related to failure in following organizational procedures. Due to the lack of professional training under the emergency situation, the captain and crew members made wrong decisions and failed to take the proper actions when the emergency emerged. In addition, the violations played an important role in the probability of accidents as all the three accidents related to the deliberate violations of rules and regulations, including the routine violations and exceptional violations. Furthermore, the consequences of the exceptional violations are unpredictable and often cause serious consequences.

### 4.5. Practical Implications

This research explores the underlying causes of three maritime accidents by employing the HFACS-MA model, and the results indicate that the HFACS-MA model is applicative in analyzing the maritime accidents. In addition, we conducted a comparative analysis on the accident causes, attempting to explore the similarities and differences about the accident causes under the similar social context. Furthermore, the demographic of the causation factors were computed, and the key categories associated with the three accidents were identified.

Regarding the three accidents, the main focus concentrated on establishing the corresponding legislations, policies, regulations, and safety culture within the maritime industry. Firstly, the legislations that related to maritime safety should be established and improved, which attempts to provide guidelines for maritime safety. Secondly, the standard procedures should be established and improved, including safety management system and rescue communications, and the in-depth risk assessment should be conducted routinely. Thirdly, the crew members on board should receive regular safety training to improve their safety knowledge and safety awareness and finally reduce unsafe acts. In addition, the rescue workers should be properly trained to improve the rescue operations level. Fourthly, it is significant to improve the meteorological warning capability and establish the information distributing platform of meteorological warning. Consequently, this research contributes to safety management and policy development in the maritime industry at different levels.

### 4.6. Limitations

Some materials of this research come from news articles, and one of the limitations is the inadequate data due to the translation obstacle of local language in Korea and Thailand when collecting evidence, which limits the depth of conclusions that we can draw. Furthermore, there are still many unresolved questions associated with the three accidents, such as the path of each accident and the risk assessment of human factors. In addition, this research is limited to accidents caused by Cruise ship and Ro-Ro passenger vessel only. Therefore, if the scope of research is expanded to multiple types of vessels, it is possible to establish effective maritime safety measures for maritime safety. Finally, HFACS was the only method used in this research. While this research was practical, it might be helpful to compare different analytical methods such as 2–4 Model [47], AcciMap [48], STAMP [49], and FRAM [50].

## 5. Conclusions

In this paper, the HFACS-MA model is introduced to explore the underlying causes of Chinese Eastern Star, Korean Sewol, and Thai Phoenix ferry accidents, indicating that the HFACS-MA model is applicable for maritime accident analysis. A comparative analysis on the accident causes of the three accidents was conducted, and the demographic of the causation factors were computed, and additionally the key categories were identified. Consequently, the research can increase support for HFACS-MA and its application, and provide valuable information for safety management and policy development in the maritime industry at different levels.

At the level of external factors, it is important to improve the legislation in the maritime industry, especially in the Eastern Star accident and Sewol accident. At the level of organizational influences, the defects in the organizational process are the key contributing factors for the three accidents, and additionally the Eastern Star accident and Sewol accident emphasize the defects in the asset management. At the level of unsafe supervision, the inadequate supervision and violations in supervision are the primary reasons for the three accidents, and it highlights the importance of adequate safety training for maritime safety. Regarding the precondition for unsafe acts, the physical environment factors featured remarkably in the three accidents, and meanwhile communication (ships and VTS) play an important part in coordination and accident rescue. At the level of unsafe acts, it emphasizes the importance of the following factors: the decision errors and violations. Efforts to reduce unsafe acts should concentrate some critical HFACS categories at the lower levels.

Although this research analyzes the causes of three accidents in detail, there are still many unresolved questions associated with the three accidents, such as the path of each accident and risk assessment of human factors. At the same time, this research provides some new insights to encourage further research to establish effective measures for national and international maritime safety.

## Figures and Tables

**Table 1:** List of abbreviations involved in this paper.

Abbreviations	Full Names
IMO	International Maritime Organization
HFACS	Human Factors Analysis and Classification System
MSA	Maritime Safety Administration
SMS	Safety Management System
CESC	Chongqing Eastern Steamship Company
KRS	Korean Register of Shipping
KSA	Korea Shipping Association
KCG	Korea Coast Guard
RMA&PA	Regional Maritime Affairs & Port Administration
CSIB	Chongqing Ship Inspection Bureau
WSIB	Wanzhou Ship Inspection Bureau
YRNA	Yangtze River Navigation Administration
VTS	Vessel Traffic Service
PMO	Phuket Marine Office

**Table 2:** A brief description of the three maritime accidents.

Information	Eastern Star	Sewol	Phoenix
Accident time	At 21:30 on 1 June 2015	At 08:58 on 16 April 2014	At 17:45 on 5 July 2018
Accident location	Yangtze River of China	Donggeochado of South Korea	Coral Island of Thailand
The number of deaths	442	304	47
The type of ship	Cruise ship	Ro-Ro passenger vessel	Cruise ship
The capacity of the ship	534 passengers and 50 crew members	921 passengers, 35 crew members, 180 vehicles, and 154 regular cargo	120 persons
Construction and modification	Initially constructed in 1994 and renovated in 1997, 2008, and 2015	Initially constructed in 1994 and renovated in 2013	Originally constructed in 2016
Information about the persons or cargo	403 passengers, 46 crew members, and 5 personnel from a travel agency	325 highschool students, 14 teachers, 108 other passengers, 29 crewmembers, and 2142.7 tons of cargo	89 passengers and 12 crew members
The registered owner of the ship	CESC	Chonghaejin	TC BLUE DREAM
Information about Passengers	Most passengers were older adults from a travel agency	Most passengers were students from the Danwon high school	Most passengers were Chinese who travel to Phuket

**Table 3:** A concise description of categories involved in the five levels of the HFACS-MA framework.

HFACS Level	Error Categories	Description
External factors (Level 1)	Legislation gaps	The defects of the current rules or guidelines that provides guidance for the maritime industry and the related organizations [28,41].
	The deficiencies in the administration	The defects of the relevant authorities in performing the current rules or guidelines, or their oversights in implementing their tasks [28].
	Flaws in design	The flaws in design or usability of equipment or materials used by crew or VTS, which are obstacles to make full use of equipment to perform tasks [42].
	Others	Some factors presented in the accident investigation report that do not belong to the above categories [8].
Organizational influences (Level 2)	Asset management	The management, distribution, and maintenance of the organizational assets such as human resources, equipment, and financial resources [28].
	Organizational climate	The working climate involved in the organization, which refers to culture, management structure, and policy [28,43].
	Organizational process	This category focuses on procedures, formal processes, and the organizational surveillance. Procedures refer to objectives, standards, and documentation. The organizational surveillance includes risk management, development, and application of safety programs. The SMS of the maritime industry is attributed to this category [11].
Unsafe supervision (Level 3)	Inadequate supervision	The factors that failed to track qualifications and provide oversight, guidance, and training, leading to unsafe conditions [11,28].
	Planned inappropriate operation	The failure in risk management, operational planning, crew arrangement, etc. [43].
	Failure to correct known problems	The supervisors did not redress the known defects in individuals, documents, equipment, procedures or training and allow these deficiencies to continue [11,28].
	Violations in supervision	The existing regulations, guidance, and operating instructions are neglected deliberately by supervisors when performing their duties, resulting in unsafe situations [28,43].
Preconditions for unsafe acts (Level 4)	Physical environment	This category refers to the factors of natural environment, including temperature, lighting, weather, wind, visibility, and currents.
	Technological environment	This category refers to failures in usability of the devices and instruments, bridge design, and checklist layouts [11].
	Adverse mental states	This category includes adverse mental conditions such as mental fatigue, inappropriate motivation, self-complacence, and lack of concentration [28,43].
	Adverse physical conditions	This category includes acute medical, pharmacological and physiological conditions which are known to have a negative effect on performance [11,43].
	Physical or mental limitations	This category refers to lack of knowledge, time, talent, and skill to perform a task [11,34].
	Resource management	This category refers to the management and usability of the relevant technical, human, and material resources that are required to ensure the safe navigation of the ship, including navigation plans, maps, electronic equipment, etc. In addition, teamwork, communication, and coordination are involved in this category [43].
	Readiness for the task	This category refers to the physical or mental reasons that individuals are not ready for performing the tasks. For instance, the use of alcohol or medications [43].
	Communication (ships and VTS)	Communications among the ships are necessary, in addition, failures in communications among ships and with VTS are the main causes of maritime accidents.
Unsafe acts (Level 5)	Skill-based errors	Skill-based errors occur when there exist flaws in memory, attention, and technique, and these errors are recognized to be mechanical behaviors [44].
	Perception errors	This category is caused by perception problems such as visual sense, auditory sense, and attention problems [44], and wrong decisions are made due to false perception.
	Decision errors	This category refers to designed and goal-intended behaviors aiming to achieve the goals, yet these behaviors are improper or inadequate. Decision errors include three subcategories: inadequate choices, procedure errors, and errors in processing the problem [28,44].
	Routine violations	Routine violations that are customarily condoned by supervising authorities are habitual behaviors [45].
	Exceptional violations	Exceptional violations often result in serious consequences and are not condoned by the management authority [43,44].

**Table 4:** The detailed list of the causation factors associated with the Eastern Star accident.

Code	Accident Causes
EC1	The necessary devices required by the IMO were not mandatory for Chinese vessels.
EC2	There was not a specific law to defend against climate disasters.
EC3	The accident information reporting procedures required by government were cumbersome.
OC1	The CESC renovated the ballast tank and water tank of Eastern Star without review.
OC2	The beds had not been fixed for a long time, and the cabin door was not equipped with a weathertight closing appliance as required.
OC3	The CESC did not establish a system to monitor and manage ships.
SC1	The CSIB and WSIB did not strictly inspect the ship according to the requirements.
SC2	The CESC tacitly consented to the Eastern Star’s adventurous voyage in dangerous environment at night.
SC3	The routine inspection from CESC was not serious.
SC4	The Yueyang MSA did not seriously implement the dynamic tracking and monitoring of passenger ships.
SC5	The YRNA granted the water transportation license without strict inspection.
PC1	The Eastern Star encountered a squall line system accompanied by a strong convective weather, tornadoes as well as torrential rain.
PC2	The Eastern Star and other five ships were involved in the storm at the same time, but the communication among ships could not work properly due to the strong storms.
PC3	The weather warning issued by MSA was not timely.
PC4	Most passengers were elderly people with limited mobility, and most of them were sleeping when the Eastern Star capsized.
AC1	The CESC illegally hired unqualified personnel to renovate the ship.
AC2	The captain failed to detect the ship’s fallback state as early as possible due to the navigation at night.
AC3	When the ship was about to capsize, the captain neither issued a distress message nor evacuated the passengers on board.
AC4	The status was not verified in time when Eastern Star disappeared from the positioning system.

**Table 5:** The detailed list of the causation factors associated with the Sewol accident.

Code	Accident Causes
EK1	The maximum service life of passenger ships went from 20 years to 30 years.
EK2	There was no clear regulation on restricting the improper renovation of ships, especially expanding the height.
EK3	The information sharing issue among supervising authorities lacked a legal basis.
EK4	Incheon RMA&PA approved the forged document about the renovations of Sewol submitted by Chonghaejin.
OK1	The Sewol was illegally renovated.
OK2	The KCG lacked standard procedures for rescue communications.
OK3	The Chonghaejin employed more informal workers than regular ones.
OK4	The KSA did not check overload strictly.
SK1	The Cheonghaejin did not provide adequate training for informal workers on the Sewol.
SK2	The KCG did not provide professional training for rescuers about the capsizing of ship.
SK3	The KRS did not strictly inspect the lifeboats.
SK4	KSA allowed Sewol to set sail without strict inspection, such as the loosened lashing devices and the overloaded cargo.
SK5	The Chonghaejin grouped and arranged two people with no experience to navigate the dangerous channel.
PK1	The Maenggol Channel had a strong tidal current with low water temperature.
PK2	The steering went wrong.
PK3	One out of 44 lifeboats worked.
PK4	The communication between the crew members and VTS was not smooth.
PK5	The emergency response from seafarers was inadequate due to their poor preparedness, inadequate training, or improper understanding of their duties.
AK1	The captain and crew members instructed the passengers to stay in the cabins instead of taking the lifeboats, while they abandoned the ship and fled.
AK2	Sewol was overloaded when leaving from the port.
AK3	Sewol was not equipped with adequate ballast water required by the classification society to keep the ship balanced.
AK4	When entering the dangerous Maenggol Channel, the captain did not navigate directly but let the inexperienced helmsman grasp the steering wheel.
AK5	The inexperienced helmsman made a sharp turn.

**Table 6:** The detailed list of the causation factors associated with the Phoenix accident.

Code	Accident Causes
ET1	The business license of the manufacturer for Phoenix had expired.
ET2	The jacuzzi that was not marked on the design was added. However, the review of the Phoenix was approved by the Thailand MSA.
ET3	There was no information platform for captains, crew members, and passengers to obtain timely meteorological information.
OT1	There were insufficient lifeguards and police officers.
OT2	The TC BLUE DREAM company lacked the basic procedures for popularizing safety knowledge when the passengers boarded the Phoenix.
ST1	The TC BLUE DREAM company did not provide adequate safety training for the captain and crew members.
ST2	The PMO had regulatory oversight in wearing lifejackets all the way.
ST3	The VTS did not contact the Phoenix actively when Phoenix disappeared from the positioning system.
ST4	The port did not take effective measures to stop vessels from leaving the port in spite of a weather warning of strong winds and waves.
ST5	Phoenix’s design drawings were unqualified but illegally approved.
PT1	The Phoenix encountered a great storm with strong winds and waves.
PT2	The drainage system was substandard.
PT3	The steering of Phoenix was not responsive.
PT4	The Phoenix and VTS at Chalong dock did not have any communication when encountering the severe storm.
AT1	The captain and crew members told passengers to stay in the cabin, while they took the lifeboats without evacuating the passengers.
AT2	The Phoenix went to sea illegally in spite of the warnings of winds and waves.
AT3	Some passengers did not wear lifejackets.

**Table 7:** The demographic of the causation factors associated with the three accidents using HFACS-MA.

HFACS-MA Category	Eastern Star Accident		Sewol Accident		Phoenix Accident	
	Causation Factors	N_C_ (%)	Causation Factors	N_K_ (%)	Causation Factors	N_T_ (%)
External factors	EC1, EC2, EC3	3(15.8%)	EK1, EK2, EK3, EK4	4(17.4%)	ET1, ET2, ET3	3(17.6%)
Legislation gaps	EC1, EC2, EC3	3(15.8%)	EK1, EK2, EK3	3(13.0%)	ET3	1(5.9%)
The deficiencies in the administration	—	—	EK4	1(4.3%)	ET2	1(5.9%)
Flaws in design	—	—	—	—	—	—
Others	—	—	—	—	ET1	1(5.9%)
Organizational influences	OC1, OC2, OC3	3(15.8%)	OK1, OK2, OK3, OK4	4(17.4%)	OT1, OT2	2(11.8%)
Asset management	OC1, OC2	2(10.5%)	OK1	1(4.3%)	—	—
Organizational climate	—	—	OK3	1(4.3%)	OT1	1(5.9%)
Organizational process	OC3	1(5.3%)	OK2, OK4	2(8.7%)	OT2	1(5.9%)
Unsafe supervision	SC1, SC2, SC3	5(26.3%)	SK1, SK2, SK3, SK4, SK5	5(21.7%)	ST1, ST2, ST3, ST4, ST5	5(29.5%)
Inadequate supervision	SC1, SC3	2(10.5%)	SK1, SK2	2(8.7%)	ST1, ST2, ST3	3(17.6%)
Planned inappropriate operation	—	—	SK5	1(4.3%)	—	
Failure to correct known problems	SC2	1(5.3%)	—	—	ST4	1(5.9%)
Violations in supervision	SC4, SC5	2(10.5%)	SK3, SK4	2(8.7%)	ST5	1(5.9%)
Precondition for unsafe acts	PC1, PC2, PC3, PC4	4(21.1%)	PK1, PK2, PK3, PK4, PK5	5(21.7%)	PT1, PT2, PT3, PT4	4(23.5%)
Physical environment	PC1	1(5.3%)	PK1	1(4.3%)	PT1	1(5.9%)
Technological environment	—	—	PK2	1(4.3%)	PT2, PT3	2(11.8%)
Adverse mental states	—	—	—	—	—	—
Adverse physical conditions	—	—	—	—	—	—
Physical or mental limitations	PC3, PC4	2(10.5%)	—	—	—	—
Resource management	—	—	PK3	1(4.3%)	—	—
Readiness for the task	—	—	PK5	1(4.3%)	—	—
Communication (ships and VTS)	PC2	1(5.3%)	PK4	1(4.3%)	PT4	1(5.9%)
Unsafe acts	AC1, AC2, AC3, AC4	4(21.1%)	AK1, AK2, AK3, AK4, AK5	5(21.7%)	AT1, AT2, AT3	3(17.6%)
Decision errors	AC3	1(5.3%)	AK1	1(4.3%)	AT1	1(5.9%)
Skill-based errors	—	—	AK5	1(4.3%)	—	—
Perception errors	AC2	1(5.3%)	—	—	—	—
Routine violations	AC4	1(5.3%)	AK2, AK3	2(8.7%)	—	—
Exceptional violations	AC1	1(5.3%)	AK4	1(4.3%)	AT2, AT3	2(11.8%)

Note: “—” represents null; N_C_ represents the number of causation factors for each category in the Chinese Eastern Star accident, and the percentage number relates to all 19 causation factors; N_K_ represents the number of causation factors for each category in the Korean Sewol accident, and the percentage number relates to all 23 causation factors; and N_T_ represents the number of causation factors for each category in the Thai Phoenix accident, and the percentage number relates to all 17 causation factors.
